# Transcriptomic analysis reveals vacuolar Na^+^ (K^+^)/H^+^ antiporter gene contributing to growth, development, and defense in switchgrass (*Panicum virgatum* L.)

**DOI:** 10.1186/s12870-018-1278-5

**Published:** 2018-04-10

**Authors:** Yanhua Huang, Xin Cui, Huifang Cen, Kehua Wang, Yunwei Zhang

**Affiliations:** 1 College of Agriculture, China Agricultural University, Beijing, People’s Republic of China; 2 College of Animal Science and Technology, China Agricultural University, Beijing, People’s Republic of China; 3 National Energy R&D Center for Biomass (NECB), Beijing Sure Academy of Biosciences, Beijing, People’s Republic of China

**Keywords:** Switchgrass, *PvNHX1*, RNA-seq, Growth and development, Plant defense

## Abstract

**Background:**

Intracellular Na^+^ (K^+^)/H^+^ antiporters (NHXs) have pivotal functions in regulating plant growth, development, and resistance to a range of stresses. To gain insight into the molecular events underlying their actions in switchgrass (*Panicum virgatum* L.), we analyzed transcriptomic changes between *PvNHX1*-overexpression transgenic lines and wild-type (WT) plants using RNA sequencing (RNA-seq) technology.

**Results:**

The comparison of transcriptomic data from the WT and transgenic plants revealed a large number of differentially expressed genes (DEGs) in the latter. Gene ontology (GO) and KEGG pathway analyses showed that these DEGs were associated with a wide range of functions, and participated in many biological processes. For example, we found that PvNHX1 had an important role in plant growth through its regulation of photosynthetic activity and cell expansion. In addition, PvNHX1 regulated K^+^ homeostasis, cell expansion and pollen development, indicating that it has unique and specific roles in flower development. We also found that transgenic switchgrass exhibited a higher level of transcription of defense-related genes, especially those involved in disease resistance.

**Conclusion:**

We showed that *PvNHX1* had an important role in plant growth and development through its regulation of photosynthetic activity, cell expansion, K^+^ homeostasis, and pollen development. Additionally, *PvNHX1* overexpression activated a complex signal transduction network in response to various biotic and abiotic stresses. In relation to plant growth, development, and defense responses, *PvNHX1* also had a vital regulatory role in the formation of a series of plant hormones and transcription factors (TFs). The reliability of the RNA-seq data was confirmed by quantitative real-time PCR. Our data provide a valuable foundation for further research into the molecular mechanisms and physiological roles of NHXs in plants.

**Electronic supplementary material:**

The online version of this article (10.1186/s12870-018-1278-5) contains supplementary material, which is available to authorized users.

## Background

Cells depend on the homeostatic maintenance of pH within specific cellular compartments to ensure optimal conditions for metabolic and enzymatic processes as well as for protein structure and function. Among the many molecular players, Na^+^(K^+^)/ H^+^ exchangers (NHXs) appear to be particularly important for the establishment and maintenance of optimal ion and pH gradients, which are essential for cell function and development [1]. In plants, NHX antiporters appeared early in evolution and belong to the CPA1 protein family, which contains many monovalent cation/ H^+^ antiporters that contribute to cellular pH, and Na^+^ and K^+^ homeostasis [2]. NHXs catalyze the electroneutral exchange of Na^+^ or K^+^ for H^+^ using the electrochemical H^+^ gradient to direct inward movement of Na^+^ or K^+^ in exchange for luminal H^+^ [3].

NHXs are ubiquitous in plants and are believed to have pivotal functions in regulating responses to salt stress [4], cold tolerance [5], drought tolerance [6], and disease resistance [7]. Recently, novel functions of NHX-type Na^+^/H^+^ antiporters were identified, including roles in cell expansion, cell volume regulation, flower development, stomatal conductance, protein processing, and vesicular trafficking [8–10]. However, the molecular mechanisms underlying these functions remain poorly understood. The recent advances in next-generation sequencing have enabled genome-wide scale and transcriptome-level computational analyses. RNA-seq technology is a powerful method to analyze the expression of genes at the transcriptome level and will provide a better understanding of the mechanisms underlying NHX function.

Switchgrass (*Panicum virgatum* L.) is a member of the Poaceae family and is a warm-season C4 perennial grass native to the U.S. It has been increasingly exploited as a dedicated bioenergy crop because of its valuable characteristics [11]. However, because it is an outcrossing and polyploid species, conventional breeding strategies to improve commercial varieties are severely restricted [12]. Fortunately, production of transgenic lines with modification of functional genes related to growth and resistance have been found to enable production of improved varieties [13–16]. For example, the successful expression of the enzyme gene *PvNHX1* was shown to promote plant growth and increase resistance to salt stress in switchgrass [16].

In this study, we performed an RNA-seq analysis to compare the transcriptomes of wild-type (WT) and transgenic plants overexpressing the *PvNHX1* gene in order to gain more insight into the function of NHXs in plants. This study has produced more information on the changes in the transcriptome in response to the overexpression of an *NHX* gene and sheds light on molecular mechanisms underlying the functions of NHXs in switchgrass. Our data provide valuable information on the potential roles of NHXs in plants.

## Methods

### Plant material and RNA extraction

Switchgrass (*P. virgatum* ‘Alamo’) was used in this study. This cultivar was originally collected in Texas (25°50′ N-36°30′ N), and we purchased seeds from Ernst Conservation Seeds (Meadville, Pennsylvania) in 2010. Switchgrass callus generated from mature seeds was transformed with a Ubi1301 binary vector (provided by the Sinogene Scientific Company) harboring a *PvNHX1* overexpression cassette using the *Agrobacterium*-mediated transformation method described previously [16]. Wild-type (WT) and transgenic switchgrass plants were grown in plastic pots containing a mixture of soil: vermiculite: humus [1:1:1 (*v*/v/v)] under the same greenhouse environment (16 h/8 h light/dark cycle) in Beijing. RNA-seq analysis was carried out using three independent transgenic lines (L1, L3, L8) and three WT plants (WT1, WT2, WT3).

When the plants reached the reproductive 1 (R1) stage (the emergence of the inflorescence from the boot stage) [17], the mature leaves of internode 3 (I3) from six independent plants were pooled for RNA extraction and frozen in liquid nitrogen for later qRT-PCR analysis.

Total RNAs were extracted from switchgrass leaves using the TRIzol reagent method (Invitrogen, Carlsbad, CA, USA). A NanoPhotometer® (IMPLEN, CA, USA) and a Bioanalyzer 2100 system (Agilent Technologies, CA, USA) were used to quantify and check the quality of the RNA samples.

### Library construction and sequencing

A total of 3 μg RNA per sample was used for sequencing library construction. Libraries were generated using NEBNext® Ultra™ RNA Library Prep Kit for Illumina® (NEB, USA) following the manufacturer’s instructions. Briefly, poly (A)-containing mRNA was purified from total RNA using poly-T oligo-attached magnetic beads (Illumina, San Diego, CA, USA). Then, the mRNA was broken into short fragments by a fragmentation buffer (Ambion, Austin, TX, USA); the fragments were used as templates for cDNA synthesis. First-strand cDNA was synthesized using random hexamer-primers and M-MuLV reverse transcriptase (RNase H). Second strand cDNA synthesis was performed using DNA Polymerase I (New England Biolabs) and RNase H (Invitrogen). Poly(A) sequences were added to the 3′ ends of cDNA fragments and sequencing adaptors with hairpin loop structure were ligated to the cDNA ends. Suitable fragments (150–200 bp) were selected by agarose gel purification and enriched by PCR amplification. Finally, the PCR amplicons were purified using magnetic beads (Illumina) and dissolved in EB solution to generate the sequencing libraries. Library quality was assessed on the Agilent 2100 Bioanalyzer.

### Quality control and sequence assembly

Raw reads obtained from sequencing were filtered to remove adaptor sequences, empty reads, and low quality sequences with ‘N’ percentage over 10%. We then calculated the Q20, Q30, GC-content, and sequence duplication levels of the clean data. All the downstream analyses were based on clean reads with high quality. The retained high-quality reads were mapped to the switchgrass reference genome sequence, *P. virgatum* v1.1 (http://www.phytozome.net/panicumvirgatum; accessed 30 November 2015) using TopHat v2.0.12 [18]. This step generates a database of splice junctions based on the gene model annotation file and gives a better mapping result than other non-splice mapping tools. An index of the reference genome was built using Bowtie v2.2.3 [19].

### Differential gene expression analysis

To quantify the abundance of transcripts, all reads from samples were mapped onto the reference transcriptome by HTSeq v0.6.1 [20]. We used the FPKM (fragments per kilobase of transcript sequence per million base pairs sequenced) algorithm to normalize gene expression abundances in each library. A differential expression analysis was performed using the DESeq R package (1.18.0) using pairwise comparisons [21]. The resulting *P*-values were adjusted using Benjamini and Hochberg’s method for controlling false discovery rates [22]. Genes with an adjusted *P*-value < 0.05 were categorized as differentially expressed.

### Gene ontology enrichment and pathway analysis

To identify putative functions of differentially expressed genes (DEGs), we performed functional annotation using a BLASTx search of two databases: Gene Ontology (GO), and Kyoto Encyclopedia of Genes and Genomes (KEGG). GO and KEGG enrichment analyses provide all GO terms or pathways significantly enriched in DEGs in comparison to the transcriptome background. GO enrichment was performed using GOseq R package [23], and the *P*-values were calculated using Benjamini and Hochberg’s method [22]. We considered a corrected *P*-value < 0.05 as a significantly enriched GO term.

In the KEGG enrichment analysis, the cellular metabolism, biochemical pathway, and potential biological behavior of DEGs were examined. KOBAS 2.0 (KEGG Orthology Based Annotation System, v2.0) software was used to test the statistical enrichment of DEGs in KEGG pathways [24]. We selected a corrected *P*-value < 0.05 as a threshold to determine significant enrichment of the gene sets.

### Novel transcript prediction

The assembled transcripts were compared with the annotated genomic transcripts from the reference sequences to identify novel transcribed regions. The coding potential calculator (CPC: http://cpc.cbi.pku.edu.cn/) was used to assess the protein-coding potential [25].

### qRT-PCR assays

Validation of RNA-seq results was carried out using quantitative real-time PCR (qRT-PCR) analysis. Primers specific to selected transcripts were designed using Premier 5.0 software (Premier Biosoft Int., Palo Alto, CA, USA). Switchgrass *ubiquitin-1* gene (*PvUBQ1*) (GenBank accession number: FL899020) was used as the internal control and was amplified using primers *PvUBQ1*-F and *PvUBQ1*-R. The qRT-PCR was performed using three biological replicates and three independent technical replicates for each sample. Gene expression levels were calculated using the 2^-ΔΔCt^ method [26]. The normalized values of relative expression and FPKM values were calculated by log_2_, respectively, and the values were used to analyze the correlation between qPCR and RNA-seq results. The primer sequences used for qRT-PCR are listed in Additional file 1.

## Results

### Morphological characterization of transgenic plants

**Fig. 1:**
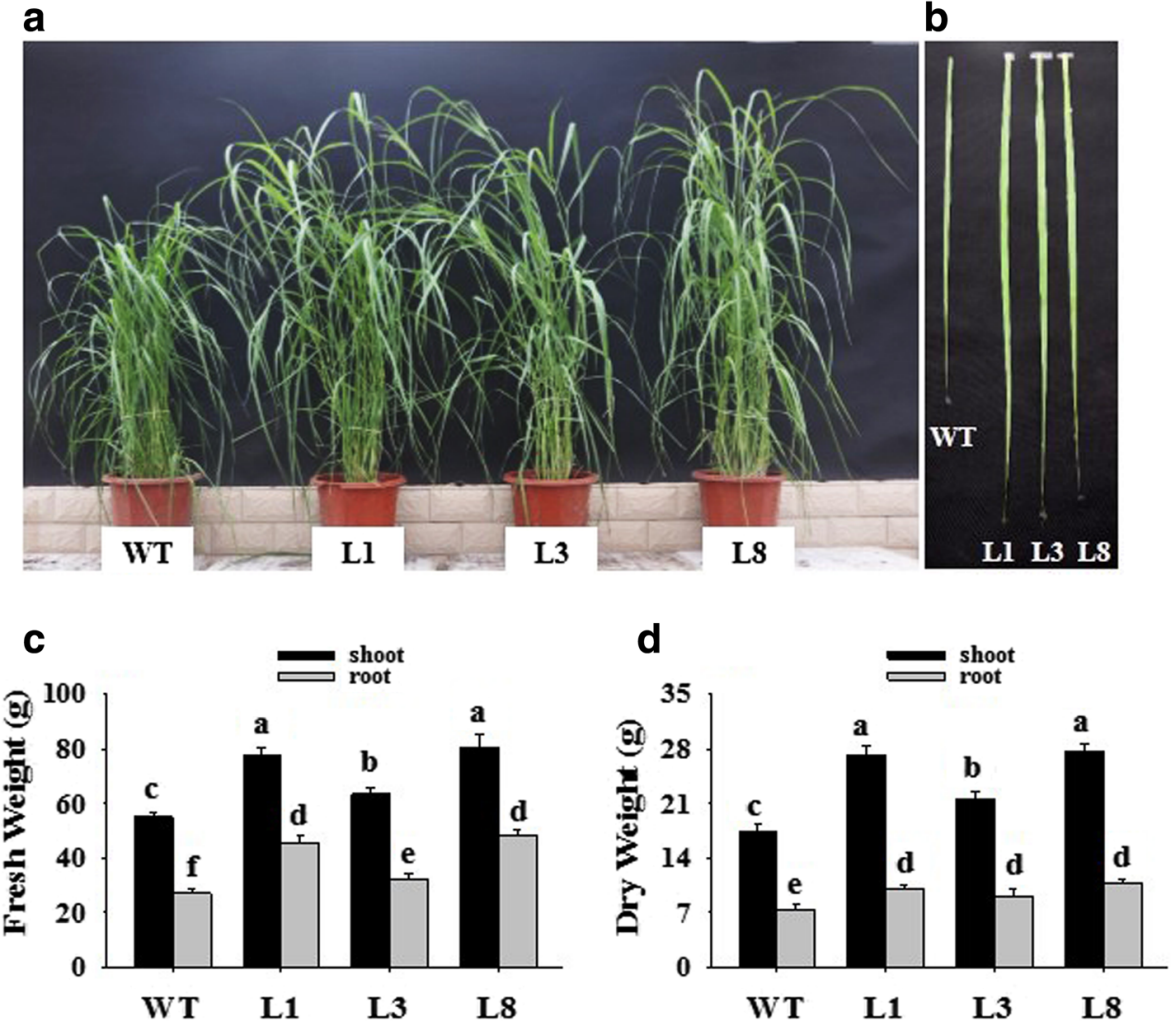
Phenotypes of transgenic *PvNHX1* switchgrass compared with WT plants. **a** Phenotypes of whole plants. **b** Leaf lengths and leaf widths in fully expanded leaves. Statistical analysis of fresh weight **c** and dry weight **d** of shoots and roots. WT: wild type; L1, L3 and L8: different transgenic lines overexpressing *PvNHX1*.The data shows the mean ± S.E. of triplicate experiments. Columns with different letters indicate significant differences at *P* < 0.01 (Duncan’s test)

Transgenic switchgrass overexpressing *PvNHX1* showed better growth and development performance than WT plants under the same greenhouse environment (Fig. 1). The transgenic plants had longer shoots, larger stem diameters, and longer leaf blade lengths and widths [16]. Based on the measurement of fresh and dry weights, the three transgenic lines showed a significantly higher biomass than the WT plants (*P* < 0.01). As shown in Fig. 1c and d, root fresh weight and shoot dry weight in the transgenic lines were 1.55-fold and 1.46-fold, respectively, greater than in the corresponding tissues of WT plants. These greenhouse phenotypes may vary under field experiments; field evaluations of yield and related traits will be performed in a future study.

### Illumina sequencing and assembly

In total, 6.60 million raw reads were generated from control samples and 6.89 million raw reads were generated from transgenic samples. The average Q20 and Q30 levels and GC-rich contents of the six samples were 97.46%, 93.52%, and 56.66%, respectively (Table 1). We obtained approximately 40.48 million total reads, of which approximately 39.66 million passed the Illumina quality filtering threshold, yielding a quality rate of over 97.98%. This result indicated that the throughput sequencing was sufficiently accurate to allow further analysis.

**Table 1:** Summary statistics of Illumina transcriptome sequencing

	wild-type (WT)			transgenic line (TG)		
	WT1	WT2	WT3	L1	L3	L8
Raw Reads	74,371,174	62,686,086	61,045,884	60,601,736	68,477,486	77,588,252
Clean Reads	72,863,350	61,435,034	59,755,422	59,183,690	67,221,546	76,144,362
Clean Bases	10.93 G	9.22 G	8.96 G	8.88 G	10.08 G	11.42 G
Error (%)	0.01	0.01	0.01	0.02	0.01	0.01
Q20 (%)	97.6	97.72	97.74	96.4	97.66	97.65
Q30 (%)	93.85	94.12	94.14	91.02	93.99	93.97
GC (%)	57.03	58.21	57.17	54.47	55.94	57.16

Reference-based transcriptome assemblies of RNA-seq data were performed using the reference switchgrass genome sequence. A total of 74.2% for WT plants and 73.9% for transgenic plants were mapped to the reference genome. Thus, there was no significant difference between transgenic and WT plants in the proportion of reads mapped to the reference genome (*p* < 0.01). A summary of the assembly statistics is provided in Additional file 2. These reads were then used for reference guided assembly and differential expression analysis.

### Differentially expressed genes (DEGs) analysis

**Fig. 2:**
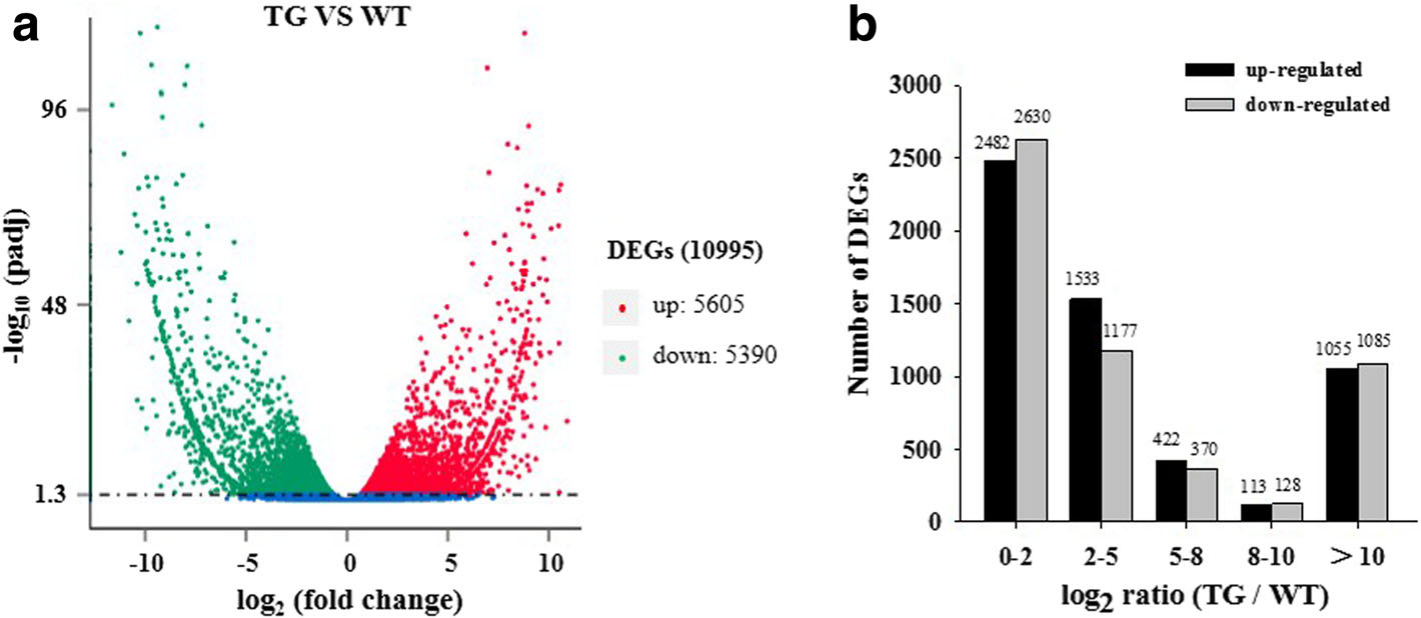
Identification of DEGs between transgenic and WT plants. **a** Volcano plot of the DEGs. **b** Statistics of genes from different expression levels. DEGs were filtered using adjusted *P*-value < 0.05 as the threshold. Red spots represent up-regulated DEGs, and green spots indicate down-regulated DEGs. Blue spots represent contigs that did not show obvious changes

The aligned reads were used to measure the relative abundances of the transcripts. A total of 10,995 differentially expressed genes (DEGs) were identified from comparison of transgenic and WT transcriptomes: 5605 transcripts showed upregulation and 5390 transcripts showed downregulation (Fig. 2a). The abundance of the different DEGs is shown in Fig. 2b. Expression of 3173 DEGs showed a large change (absolute value of log_2_ ratio ≥ 5): 1590 of these DEGs were upregulated and 1583 were downregulated.

We identified many upregulated DEGs related to photosynthesis. Genes involved in chlorophyll biosynthesis (Pavir.J25629), chloroplast development (Pavir.J24058), photosynthetic electron transport (Pavir.J30556), light-signal transduction (Pavir.Eb03789), carbon dioxide fixation (Pavir.J18292), and NAD/NADP binding (Pavir.Ia04122) were significantly upregulated (Table 2). Some transcripts with significant upregulation in transgenic plants were involved in cell division and cell elongation processes (Additional file 3). Genes involved in plant responses and adaptation to stress, such as heat (Pavir.Aa00547, Pavir.J01404), salt (Pavir.Ha00186, Pavir.Ea00535), drought (Pavir.J16055), oxidative (Pavir.J40048, Pavir.Ba01869), heavy metal (Pavir.Ba00376, Pavir.Ha00320), and certain herbicides (Pavir.Ea01215, Pavir.Ia04853), were significantly upregulated in transgenic plants compared to WT plants (Additional file 4). The expression of ion transport genes (Pavir.Eb03651, Pavir.J05404, and Pavir.Aa03191), small molecule transport genes (Pavir.Fa02242, Pavir.Eb02833, and Pavir.Ia02157.), and metal transport genes (Pavir.J06378, Pavir.Ia01399, and Pavir.J38980) were also up-regulated (Additional file 5). These results suggested that *PvNHX1* plays diverse roles in regulating plant development and stress tolerance.

**Table 2:** Significantly upregulated genes involved in photosynthesis

Gene ID	log_2_Ratio (TG vs.WT)	Q value	Annotation
Chlorophyll biosynthesis and chloroplast development			
Pavir. J25629	Inf	3.55 E-55	Involved in the chlorophyll biosynthesis
Pavir. J38707	9.5381	6.72 E-37	Involved in chlorophyll b degradation
Pavir. Ea00096	8.8125	1.41 E-26	Required for proper chloroplast degradation
Pavir. J16419	5.6144	4.95 E-03	Involved in the formation of chlorophyll
Pavir. J24058	8.5303	8.61 E-30	Regulate gene encoding chloroplast
Pavir. Ba03510	6.4468	6.14 E-05	Essential for chloroplast development
Pavir. Eb03460	4.8864	2.94 E-17	Light-induced chloroplast development
Pavir. J40341	3.8929	5.78 E-05	Regulate chloroplast photosynthetic capacity
Photosynthetic electron transporter and light-signal transduction			
Pavir. J30556	Inf	1.59 E-17	Functions as an electron carrier in photosystem I
Pavir. J34572	Inf	1.14 E-40	Functions as an electron transporter
Pavir. J23449	3.0941	7.74 E-03	Electron transfer activity
Pavir. J24058	8.5303	8.61 E-30	Positive regulator of photomorphogenesis
Pavir. Eb03789	5.7887	8.07 E-12	Involved in light-signal transduction
Pavir. Ia02931	5.0042	8.51 E-04	Involved in light-signal transduction
Carbon dioxide fixation and NAD/NADP binding			
Pavir. J05582	5.0109	2.59 E-04	Eliminate the photorespiratory loss of CO_2_
Pavir. Ca00150	3.5885	3.39 E-03	Involved in reversible hydration of CO_2_
Pavir. J18292	3.4094	9.05 E-26	Involved in carbon dioxide fixation
Pavir. J30434	Inf	5.72 E-06	Glycerol-3-phosphate dehydrogenase activity
Pavir. Ia04122	3.1439	7.00 E-03	Alcohol dehydrogenase (NAD) activity
Pavir. Ia04122	3.0679	3.79 E-03	D-3-phosphoglycerate dehydrogenase

### Functional classification by gene ontology analysis

**Fig. 3:**
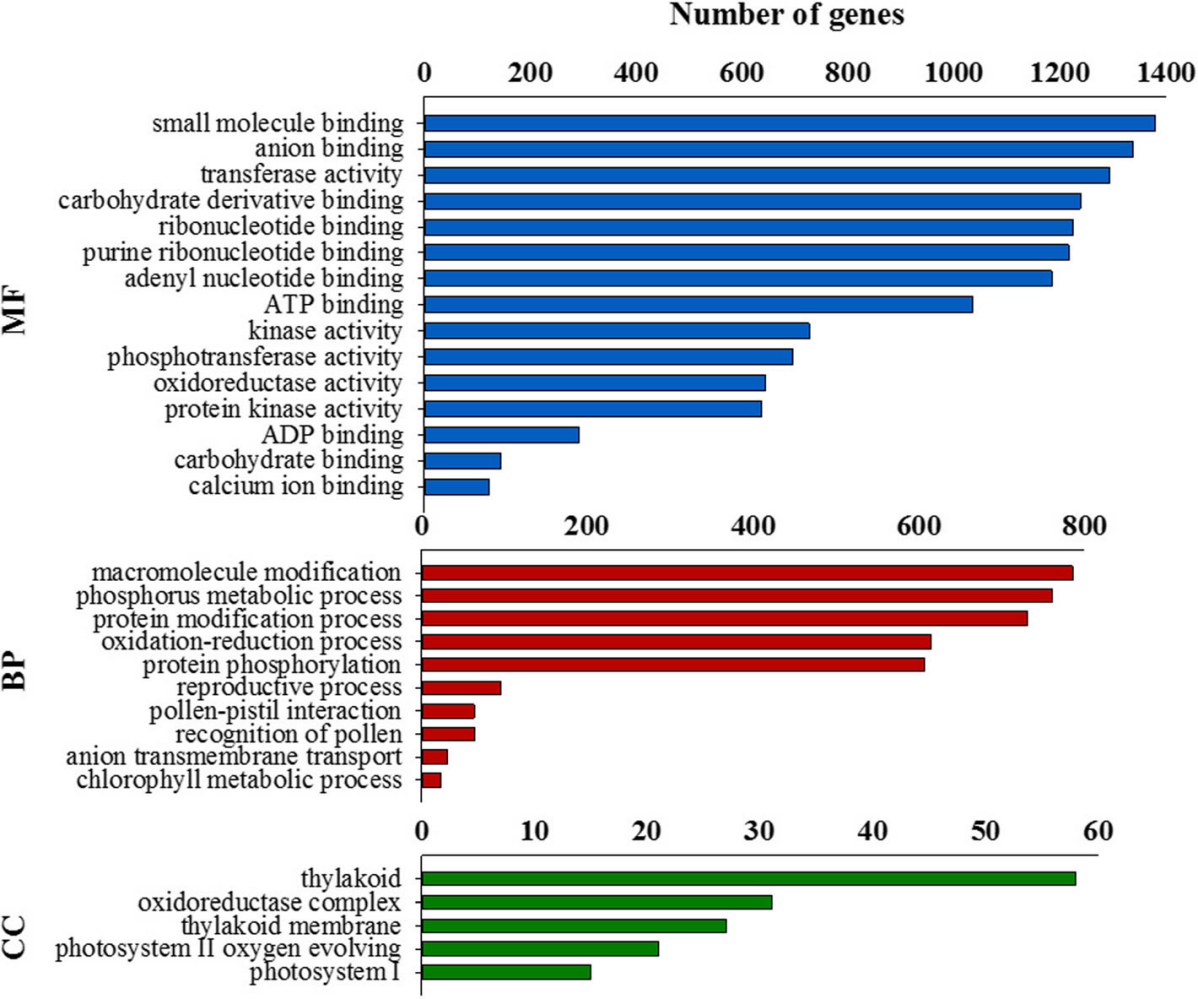
GO classifications of DEGs between transgenic *PvNHX1* lines and WT plants. GO terms were functionally classified into three GO categories: molecular function (MF), biological process (BP), and cellular component (CC). The X-axis indicates number of contigs in each category. The left Y-axis represents GO categories

To evaluate the potential functions of unigenes with significant transcriptional changes between transgenic and WT plants, we performed a GO enrichment analysis. In total, 63 GO terms were functionally classified into three GO categories, namely molecular function (35 members), biological processes (23 members) and cellular components (5 members). For molecular function, the overrepresented GO terms were small molecule binding (GO:0036094), anion binding (GO:0043168), and transferase activity (GO:0016740). In the category of biological processes, two GO terms ‘macromolecule modification (GO:0043412)’ and ‘phosphorus metabolic process (GO:0006793)’ were significantly enriched. The thylakoid term (GO:0009579; 38.2%) was the largest in the cellular components category (Fig. 3).

We further analyzed the overrepresented GO functions within each ontology. The enriched GO terms of molecular function were classified into two branches: binding and catalytic activity. This was consistent with the previous functional definition of PvNHX1. Classification of GO terms enriched in biological processes showed these were mainly related to protein phosphorylation and pollen-pistil interactions (Additional file 6). All GO terms identified in the cellular components were found to be related to plant photosynthesis.

### Pathway enrichment analysis of DEGs

**Fig. 4:**
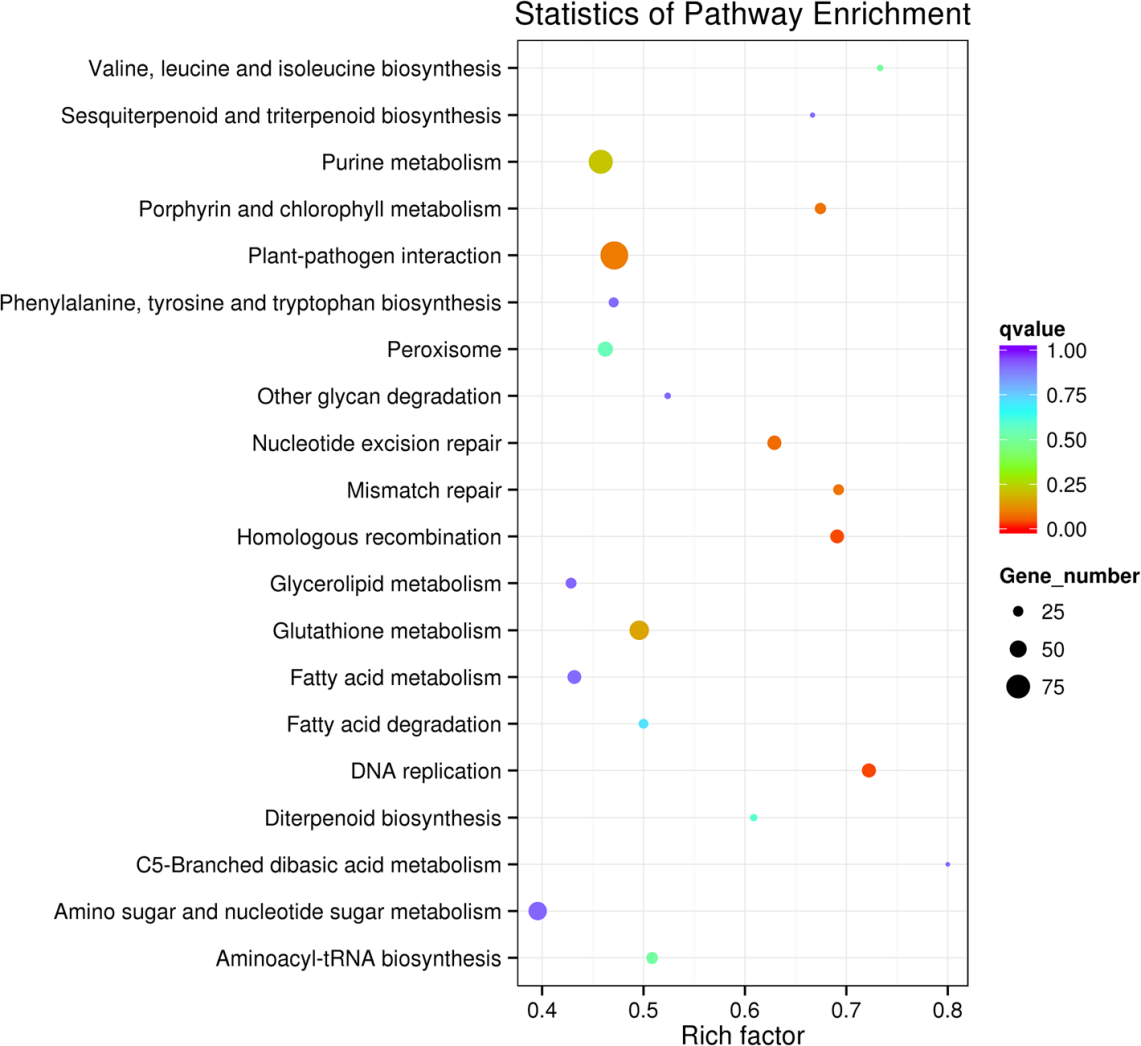
KEGG enrichment of the annotated DEGs across three comparisons. The Y-axis indicates the KEGG pathway. The X-axis indicates the Rich factor. A high q value is represented by blue, and a low q value is represented by red

**Fig. 5:**
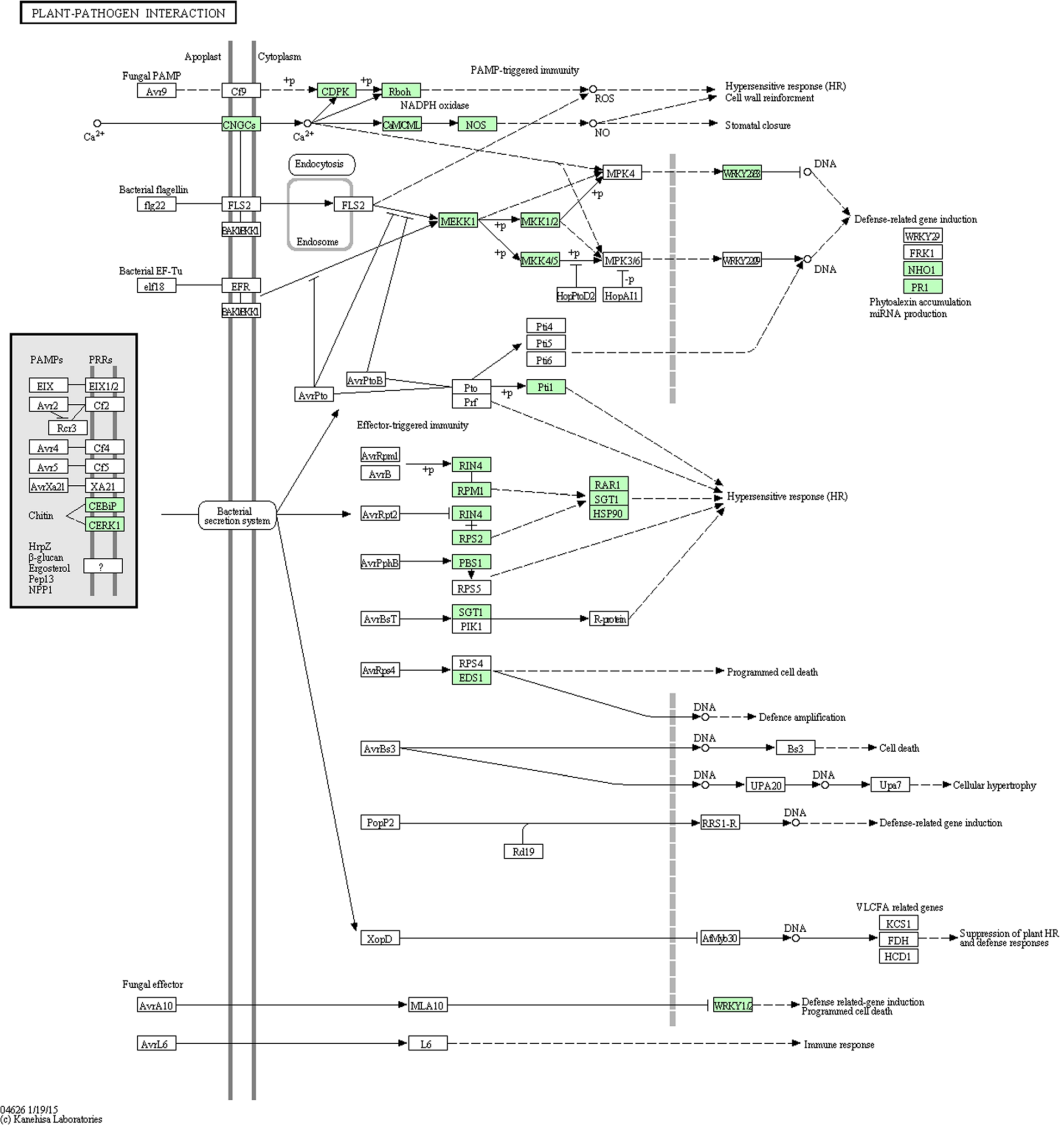
DEGs in transgenic switchgrass encode proteins involved in the plant-pathogen interaction pathway (KEGG: sita04626)

The metabolic pathways affected by *PvNHX1* overexpression were evaluated by mapping the DEGs to reference canonical pathways in the KEGG database. A total of 10,995 DEGs were mapped to 121 KEGG pathways, and 11 pathways were significantly enriched (*P*-value < 0.05). Among the significantly enriched pathways were ‘plant-pathogen interaction (KO: sita04626)’, ‘purine metabolism (KO: sita00230)’, ‘Peroxisome (KO: sita04146)’ and ‘Porphyrin and chlorophyll metabolism (KO: sita00860)’ (Fig. 4, Additional file 7). Ninety DEGs were categorized in the ‘plant-pathogen interaction’ pathway. These included signal transduction components (Ca^2+^ signaling, protein kinase, and phosphatidylinositol signal molecules), defense response proteins (calcium binding protein, serine/threonine-protein kinase, and glycerol kinase), and defense proteins against fungi or bacteria (Fig. 5). We also identified many genes encoding disease resistance proteins, such as *RPM1* (Pavir. J05404), *RGA1* (Pavir. Gb01964), *TAO1* (Pavir. Gb01964), and *RPP1* (Pavir. J20461) (Table 3). These annotations provide a valuable resource for investigating the function of *PvNHX1* in switchgrass pathogen defense.

**Table 3:** List of disease resistance genes identified in transgenic compared to WT plants

No.	Gene name	Gene ID	log_2_Ratio (TG vs.WT)	*Q* value
1	RPM1	Pavir. J04308	9.0709	2.62 E-49
2	RGA1	Pavir. Gb01964	8.2700	1.83 E-39
3	RGA2	Pavir. Fa02339	6.0500	1.62 E-42
4	RGA3	Pavir. Ba04033	4.8092	2.71 E-03
5	RGA4	Pavir. Ba02211	7.4103	9.58 E-30
6	TAO1	Pavir. Ba03591	3.4021	4.22 E-05
7	RPP1	Pavir. J20461	5.4335	4.18 E-05
8	RPP13	Pavir. Hb01356	5.8656	2.95 E-03
9	RPPL1	Pavir. Gb00467	8.0560	2.75 E-30
10	RPP13L3	Pavir. J08640	5.7515	6.25 E-05
11	RPP13L4	Pavir. Fb00106	7.8920	8.24 E-18
12	RXW24L	Pavir. Cb00342	4.9239	1.04 E-02
13	GDPDL2	Pavir. J28202	6.2089	6.28 E-07
14	At1g58400	Pavir. Ha01139	10.4920	4.06 E-68
15	At1g59780	Pavir. J11190	5.8864	1.69 E-05
16	At1g50180	Pavir. J17143	3.1030	4.78 E-02

Transgenic switchgrass overexpressing *PvNHX1* has been demonstrated to have an altered plant hormone signal transduction pathway (KO: sita04075). Auxin signaling-related genes were upregulated in the transgenic plants, including SAUR-like auxin-responsive protein gene (Pavir.Ga00493), auxin efflux carrier gene (Pavir.J25610), and auxin response factor 5 (Pavir.J32718) (Table 4). Gibberellin, salicylic acid, jasmonic acid, brassinosteroids, and abscisic acid signaling-related genes were also significantly upregulated (*q*-value < 0.01) in transgenic plants. Ethylene signaling was altered in transgenic plants, with upregulation of two genes (Pavir.Bb03119 and Pavir.Ea01573) and downregulation of two genes (Pavir.Fa00424 and Pavir.J10765). The cytokinin biosynthesis genes, Pavir.Ba01358 and APavir.J18777, were upregulated, while the cytokinin dehydrogenase 11 gene (Pavir.Fa00713) was downregulated in transgenic plants (Table 4).

**Table 4:** Genes in the plant hormone signal transduction pathway with altered expression

Hormone	Gene ID	log_2_Ratio (TG vs.WT)	Annotation
Auxin	Pavir.J19751	9.4224	Involved in auxin-activated signaling pathway
	Pavir.Ga00493	3.4397	SAUR-like auxin-responsive protein; SAUR71
	Pavir.J25610	3.4175	Auxin efflux carrier component 1b; PIN1B
	Pavir.Fb01962	2.5885	Auxin-induced in root cultures protein; AIR12
	Pavir.J32718	2.5492	Auxin response factor 5-like; ARF5
Cytokinin	Pavir.Ba01358	2.4615	Cytokinin ribosides 5′-monophosphates; LOGL9
	Pavir.J18777	2.3612	Histidine containing phosphotransfer; AHP1
	Pavir.Fa00713	−2.3029	Cytokinin dehydrogenase 11; CKX11
Gibberellin	Pavir.Cb00641	5.9078	Synthesis of gibberellin precursor; KS1
	Pavir.Aa00893	2.6192	Gibberellin-regulated protein 3; GASA3
Ethylene	Pavir.Bb03119	Inf	Acts as a regulator of ethylene signaling; EIN4
	Pavir.Ea01573	5.9235	Involved in the ethylene biosynthesis; ACO1
	Pavir.Fa00424	−3.5140	Ethylene insensitive 3-like 3 protein; EIL3
	Pavir.J10765	−3.1209	Reversion-to-ethylene-sensitivity1; RTE1
Salicylic acid	Pavir.Eb03507	7.9131	Mediating salicylic acid transcription; HBP1C
	Pavir.Ha01753	3.4826	Salicylic acid-inducible transcription; TGA21
Abscisic acid	Pavir.Ib02120	3.3525	Abscisic acid 8′-hydroxylase 1; CYP707A5
Jasmonic acid	Pavir.J01580	Inf	Jasmonate ZIM domain-containing protein; JAZ
Brassinosteroid	Pavir.Fb01473	Inf	Brassinosteroid insensitive 1 protein; BRI1

### Identification of transcription factors

**Fig. 6:**
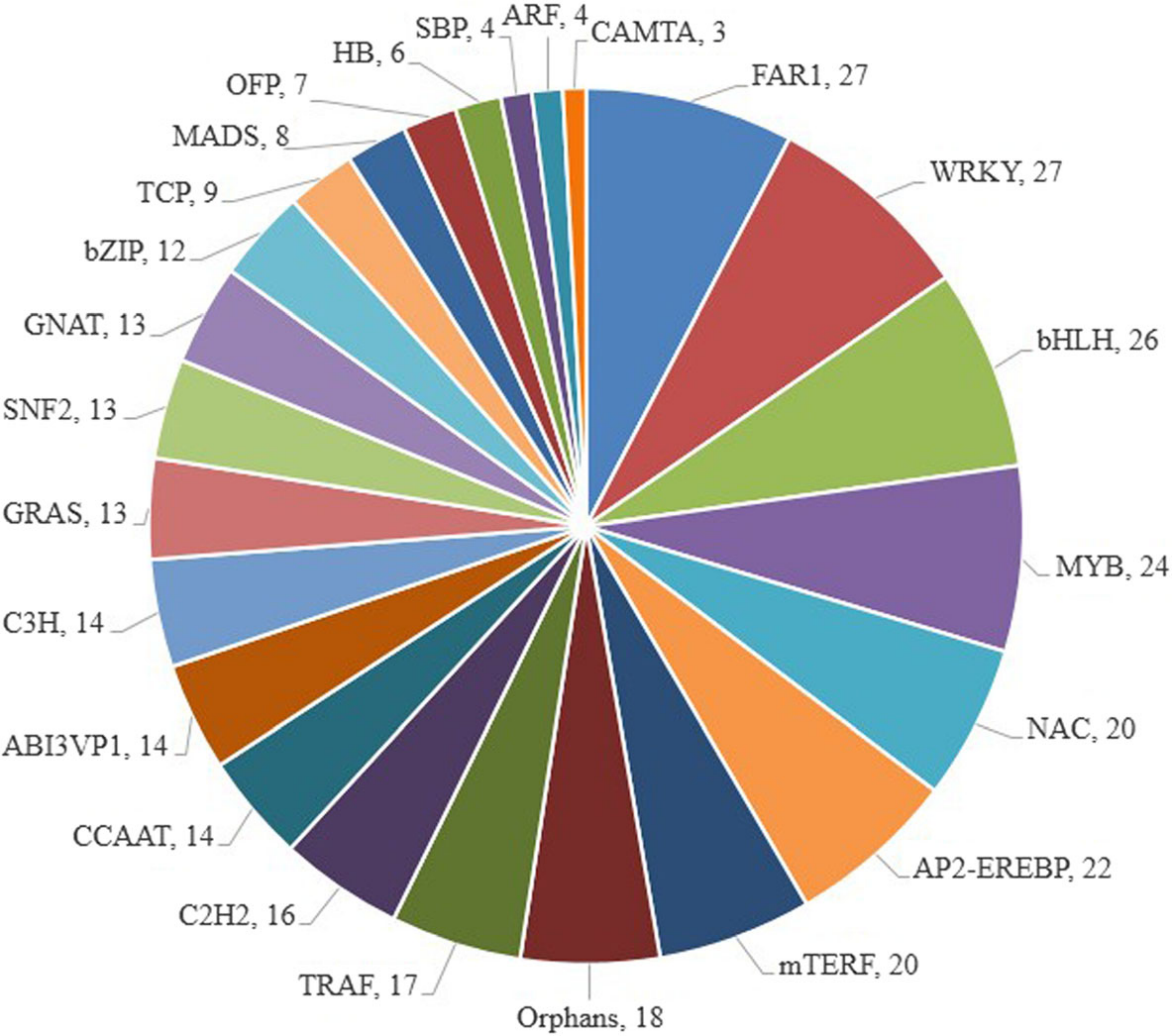
Distribution of transcription factors families in transgenic switchgrass

We performed a global transcription-factor classification of differentially expressed transcripts and identified 452 transcription factors (TFs) belonging to 59 TF families. Among these TF families, FAR1 and WRKY were most abundant, followed by bHLH (26, 5.75%), MYB (24, 5.31%), AP2-EREBP (22, 4.87%), and mTERF (20, 4.42%) (Fig. 6). Notably, all members in the SBP, HB, TCP, and CCAAT families were downregulated, and members in the CAMTA and ARF families were up-regulated. In the C3H and C2H2 families, similar numbers of up- and downregulated members were present (Additional file 8).

### Validation of gene expression profiles using qRT-qPCR

**Fig. 7:**
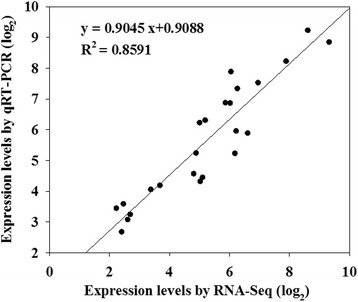
Correlations of expression levels analyzed by log_2_RNA-Seq platform with data obtained using log_2_qPCR. X-axis: log_2_RNA-Seq;Y-axis: log_2_qPCR

To confirm the accuracy and reproducibility of the Illumina RNA-seq results, 24 DEGs were selected for qPCR assays, including growth-related, resistance-related and transporters-related genes. The qPCR results for selected DEGs showed good agreement with the transcript-abundance changes determined by RNA-seq. For example, Pavir.Ia04853, glutathione S-transferase F11 (GSTF11) was upregulated 8.85-fold, and the FPKM value in RNA-seq was 9.32 (Additional file 9). A highly significant correlation was found between the qPCR and RNA-seq (R^2^ = 0.8591, *P* < 0.01), indicating the reproducibility and reliability of the RNA-seq data (Fig. 7).

## Discussion

It was previously shown that transgenic switchgrass overexpressing *PvNHX1* exhibited significantly better growth performance than WT plants (*P* < 0.05) and that the enhanced growth phenotype was associated with the expression level of the transgene in different lines [16]. Recent studies have suggested that NHXs play an important role in orchestrating plant growth by influencing the rates of photosynthetic activity [27, 28] and cell expansion [29, 30]. The results of the transcriptome analysis here are consistent with this suggestion. All GO terms identified in cellular components were related to plant photosynthesis (Fig. 3). The level of transcription of related genes involved in photosynthesis and cell expansion were significantly upregulated in transgenic lines (Table 2; Additional file 3). Other studies have shown that *NHXs* are abundantly expressed in flower organs of transgenic rice [31], *Arabidopsis* [32], mungbean [4], and cowpea [33]. Thus, NHXs would be expected to be involved in the regulation of plant flower development. Transgenic switchgrass overexpressing *PvNHX1* was found to display two distinct expression patterns in key flowering-time regulators, suggesting that the role of NHXs in flowering time was complex and did not simply act through promotion or inhibition [16]. In this study, our GO analysis indicated that DEGs in biological processes were associated with flower development, such as pollen-pistil interaction, and recognition of pollen and pollination, suggesting that NHXs might have specific roles in pollen development (Fig. 3; Additional file 6). This speculation is supported by findings from *Arabidopsis*, in which the filaments in plants with the double knockout *nhx1 nhx2* did not elongate sufficiently to position anthers at the height of the stigma. Additionally, the anthers lacked the ability to dehisce and release pollen, leading to a failure of flower set and silique formation [34]. Overall, our data point to an important role in which *PvNHX1* regulates K^+^ homeostasis, cell expansion, and pollen development in stamens, and that this homeostasis enables filament elongation and anther dehiscence to occur. Our analysis showed that the potassium channel gene *KCO2* (Pavir. J05404), a highly selective inward-rectifying potassium channel [35], and three *HAK* (high-affinity potassium transporter) genes, that exhibit potassium ion transmembrane transporter activity [36], were significantly upregulated in transgenic plants (Additional file 5). Moreover, MSP1 (Pavir.Ha01736), which is involved in cell specification during anther development and initiation of anther wall formation [37] (Additional file 3), and LecRK42 (Pavir.J33874), which is required for pollen development in *Arabidopsis* [38],

Were also significantly upregulated in transgenic plants. These results provide information on the molecular mechanisms in which NHXs participate in flower development.

NHXs were previously shown to have a key role in plant responses to abiotic stresses [4–6, 16]. Our observations here were consistent with those reports as we found that transgenic switchgrass had higher levels of transcription of related genes, for instance, Pavir.J31898 (general defense protein), Pavir.Ha00186 (response to salt stress), Pavir.Aa00547 (response to hyperosmotic and heat shock), and Pavir.J16055 (response to drought and freezing stress) (Additional file 4). We also found significant upregulation of genes related to oxidative stress (Pavir.J40048; Pavir.Ba04000; Pavir.Ba01869) and heavy metal stress (Pavir.Ba00376; Pavir.Ha00320), suggesting stress responses are activated in transgenic *PvNHX1* lines. To date, little is known about the function of NHXs in biotic stresses, such as weed stress and pathogen attack. In the present study, we found that the glutathione S-transferase genes (Pavir.Ea01215; Pavir.Ia04853), which have a significant detoxification activity against some herbicides [39], were significantly upregulated (Additional file 4). Our results also showed significant enrichment of DEGs related to metabolic pathways for plant-pathogen interactions (Fig. 4, Additional file 7). These results suggest a close correlation between NHXs and plant disease resistance. This speculation is supported by a report from tobacco (*Nicotiana benthamiana* L.), in which *NbNHX1* silencing resulted in increased sensitivity to *Phytophthora parasitica* var. *nicotianae* (*Ppn*) sensitivity, whereas ectopic expression of *NHX1* from *Salicornia europaea* or *Arabidopsis* enhanced *Ppn* resistance in tobacco [40]. Here, we sought to obtain greater insights into the molecular events underlying *NHX* activities in plant disease resistance by analyzing the level of transcription of DEGs involved in plant-pathogen interaction pathways. Our results showed that many DEGs were involved in pathways for biosynthesis of secondary signaling compounds, such as Ca^2+^ signaling, protein kinase, and phosphatidylinositol signaling (Fig. 5). Protein kinases represent an important mechanism in defense signal transduction, and have been implicated in a wide variety of plant biotic and abiotic stress responses [41]. These findings suggest that overexpression of *PvNHX1* activated a complex signal transduction network and enhanced disease resistance. In addition, the expression levels of many genes encoding disease resistance proteins were significantly upregulated in *PvNHX1*-overexpressing plants, for instance, *RPM1* [42], *RGAs* [43], *TAO1* [44], *RPP1* [45], *RPP13* [46], and *GDPDL2* [47]. We also identified many potential disease resistance genes, such as *RXW24L* (Pavir. Cb00342), *RPPL1* (Pavir.Gb00467), *RPP13L3* (Pavir.J08640), *RPP13L4* (Pavir.Fb00106), *At1g58400* (Pavir.Ha01139), *At1g59780* (Pavir.J11190), and *At1g50180* (Pavir.J17143) (Table 3). These disease resistance genes may be a valuable resource for future molecular breeding to develop plants with greater protection against multiple diseases.

Plant hormones have vital regulatory roles in plant growth, development, and defense response. These hormones can be functionally divided into growth hormones (auxins, cytokinins, gibberellins, and brassenosteroids), and stress hormones (abscisic acid, jasmonic acid, and salicylic acid) [48]. In the present study, transgenic plants overexpressing *PvNHX1* showed upregulation of five auxin signaling-related genes, such as *SAUR71* [49], *PIN1B* [50], and *ARF5* [51] (Table 4). *PvNHX1* also influenced gibberellin signaling, which is involved in the regulation of plant growth and flowering. Most genes involved in cytokinin and brassenosteroid signaling pathways were upregulated, except *CKX11* (Pavir.Fa00713), which catalyzes the oxidation of cytokinins [52], that was downregulated. Ethylene plays a crucial role in plant growth and development, and also functions in regulation of responses to various biotic and abiotic stresses. Overexpression of *PvNHX1* changed the transcription levels of many genes involved in ethylene biosynthesis and signal transduction (Table 4). In addition, many genes involved in abscisic acid, jasmonic acid and salicylic acid signaling pathways were altered, suggesting a close correlation between *PvNHX1*-induced plant defense responses and these hormone-regulated pathways. In general, TFs control differential gene expression in most major biological processes. In the present study, many TFs, such as WRKY, MYB, FAR1, and bHLH, were found to be either upregulated or downregulated in the transgenic plants (Fig. 6; Additional file 8). These results indicate that the critical roles of *PvNHX1* in plant growth, development, and defense are mediated via transcriptional regulation of related genes and/or TFs.

## Conclusions

This study provides a comprehensive overview of the regulation of transcription in transgenic switchgrass overexpressing *PvNHX1*. We identified a number of DEGs and annotated these using the GO and KEGG databases. This study demonstrated that *PvNHX1* had an important role in plant growth and development through its regulation of photosynthetic activity, cell expansion, K^+^ homeostasis, and pollen development. Focusing on the regulatory mechanisms of stress response, we found that *PvNHX1* overexpression activated a complex signal transduction network to enhance disease resistance. Many known and potential disease resistance genes were also identified and will be of use in future molecular approaches to switchgrass breeding. Our analyses also showed that overexpression of *PvNHX1* altered the transcription of hormones and TFs that have roles in the regulation of plant growth, development, and defense mechanisms. To our knowledge, this is the first report on gene expression profiling of transgenic switchgrass overexpressing *PvNHX1* using RNA-seq technology. These data will contribute to our understanding of the molecular mechanisms underlying the action of NHXs in plants, and provide important clues for further study of genes and networks that contribute to growth, development and defense responses in switchgrass.

## Additional files

Additional file 1: **Table S5.** Primer sequences used in the experiments. (DOCX 16 kb)

Additional file 2: **Figure S1.** Total mapped and unmapped RNA-seq clean reads for transgenic lines and WT plants. (PDF 119 kb)

Additional file 3: **Table S1.** Significantly upregulated genes involved in cell division in transgenic compared to WT plants. (DOCX 15 kb)

Additional file 4: **Table S2.** List of stress-responsive genes in transgenic compared to WT plants. (DOCX 15 kb)

Additional file 5: **Table S3.** Significant upregulated transporters in transgenic compared to WT plants. (DOCX 16 kb)

Additional file 6: **Figure S2.** Enriched gene ontologies in differentially expressed genes of transgenic switchgrass. Each box shows the GO term number, the *p*-value in parenthesis, and GO term. Box colors indicates levels of statistical significance: yellow = 0.05; orange = e-05; and red = e-09. (PDF 269 kb)

Additional file 7: **Table S4.** Number of genes identified from KEGG pathways. (DOCX 16 kb)

Additional file 8 Figure S3 Differentially expressed transcription factors in transgenic switchgrass. Blue bar represents down-regulated DEGs; red bar indicates up-regulated DEGs. (PDF 133 kb)

Additional file 9 Figure S4 Expression analysis of selected RNA-seq genes by qRT-PCR. FPKM (fragments per kilobase of exon per million fragments mapped) values obtained with RNA-seq and qPCR values in the analysis of selected genes. Error bars represent the standard error for three independent experimental replicates. (PDF 146 kb)

## Abbreviations

CPC: coding potential calculator
DEGs: differentially expressed genes
FPKM: fragments per kilobase of transcript sequence per million base pairs sequenced
GO: gene ontology
KEGG: Kyoto Encyclopedia of Genes and Genomes
KOBAS: KEGG Orthology Based Annotation System
qRT-PCR: quantitative real-time PCR
RNase H: M-MuLV reverse transcriptase
RNA-seq: RNA sequencing
TFs: transcription factors
WT: wild-type
