# Supplementary material for: Transcriptomic analysis reveals vacuolar Na+ (K+)/H+ antiporter gene contributing to growth, development, and defense in switchgrass (Panicum virgatum L.)
Source: BMC Plant Biol. 2018 Apr 10;18:57. doi: 10.1186/s12870-018-1278-5 (PMC5892015; doi:10.1186/s12870-018-1278-5)
Supplement: Supplementary file 1 — Table S5. Primer sequences used in the experiments. (DOCX 16 kb) [file 12870_2018_1278_MOESM1_ESM.docx]

| **Gene name** | **Gene ID** | **Forward primer (5’-3’)** | **Reverse primer (5’-3’)** |
| --- | --- | --- | --- |
| RGA2 | Pavir.Fa02339 | CCATGAGGTCCAAGGAGGTA | CTGGTGCTGGAGCCTGTAAT |
| RGA3 | Pavir.Ba04033 | GTAGCGGATGTCGAGTCCAG | ATGGCATCCTTCAGCATCTC |
| RPP13 | Pavir.Hb01356 | TCTCATCGCTCACCAAGCTA | AAGCCTCTGAAGTGGTGGTG |
| RPP13L4 | Pavir.Fb00106 | AAGTCCGTTCTCTTGCCGTA | CCGTGAGGTCGATTACCATC |
| TL1 | Pavir.Ia02326 | TGGAACTGTCTGTGGTGGTG | AGAGAAGTGGAGCGAGCAAG |
| WAK2 | Pavir.Da02121 | GAAGCTGGCGAATATCAAGC | CTCGGCTTCTCTGAAGATCG |
| COBL7 | Pavir.Ba00279 | CCGTCCAAGGTCTTCTTCAA | AGCAAGAGCAGAGCTGAGGT |
| CNR8 | Pavir.J16678 | GCTGTTTGCCTTACACTGGTC | ATTGTAGCGCCGACGAATAG |
| CNR2 | Pavir. J20246 | GTGCGTCTTCTCCTGCTTCT | CTTCTTGAGCTCGCGGTACT |
| XTH8 | Pavir.J39558 | CGAGATCGACATGGAGTTCA | TGGTGTAGGTGTGGAAGTCG |
| MSP1 | Pavir.Ha01736 | GATCTTGGATAAGCGGATGG | CACCACCTCCTCCATTGTC |
| ABCG50 | Pavir.J31898 | GCACTTACGTTCCGCAATCT | AAGAACACCAGGACGGAATG |
| GSTF11 | Pavir.Ia04853 | AGGACGTGCCATTCCAGAT | GACAGTGGTGAGGTGGTCCT |
| SKD1 | Pavir.Ea00535 | GCAACTATGTCAAGGCGTTC | CGGAGGTACTCGGTGAACTT |
| ERD15 | Pavir.J16055 | AGTGGTGGGAGCTCGTCAAG | AAGAGGTCGTCGGTGTCGAG |
| PER51 | Pavir.J40048 | GATCACTTCACCACTTCCGTC | TGAAGGTCTCCCGGTACTTG |
| GSTU17 | Pavir.Ia02931 | GAGTTCGTGGAGGAGGAGGT | GACGTACTGGACGATGACGA |
| CEST | Pavir.Eb03460 | GATGCGGCTGCTGAGTATG | CCTTCTGACTTCGCGGATATT |
| GPT2 | Pavir.Ba01179 | GGCAACACCATGAAGAGGAT | AGGTTCCAAGAATGGCAATG |
| NRAT1 | Pavir.Aa03191 | GTGCTTCAGAGTGGCAGTGA | AGCTCCAGGTCCAACATGAG |
| POR1 | Pavir.J21405 | TTGGTTCTCAGCATTCGTTG | TCGACCTCACCAGAGATGG |
| BASS1 | Pavir.Ia01399 | CTTGGCATCTCCTTCACGAT | AGTGGCATCACCGTGTATTG |
| HAK27 | Pavir. Ib03819 | TCTCCCTTGGTGGCATACTC | TGATGGGACTAAACCGAAGC |
| HAK5 | Pavir. Ea03843 | AATCGTACCAAGAGGGCTCA | ACGCCATCACCAATTACCAT |

**Table S5.** Primer sequences used in the experiments
