# Supplementary figures and images for: Transcriptomic analysis reveals vacuolar Na+ (K+)/H+ antiporter gene contributing to growth, development, and defense in switchgrass (Panicum virgatum L.)
Source: BMC Plant Biol. 2018 Apr 10;18:57. doi: 10.1186/s12870-018-1278-5 (PMC5892015; doi:10.1186/s12870-018-1278-5)

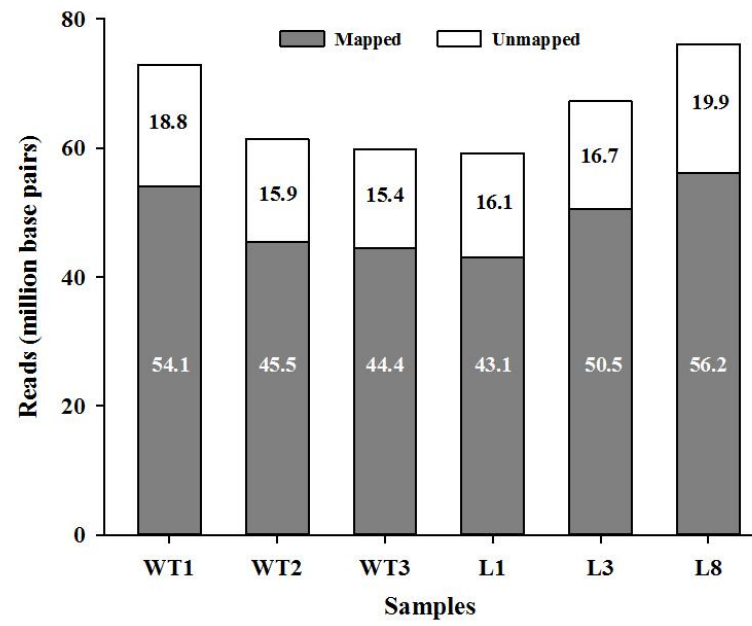

**Figure S1.** Total mapped and unmapped RNA-seq clean reads for transgenic lines and WT plants.

Supplement: Supplementary file 2 — Figure S1. Total mapped and unmapped RNA-seq clean reads for transgenic lines and WT plants. (PDF 119 kb) [file 12870_2018_1278_MOESM2_ESM.pdf]
