# Supplementary material for: Transcriptomic analysis reveals vacuolar Na+ (K+)/H+ antiporter gene contributing to growth, development, and defense in switchgrass (Panicum virgatum L.)
Source: BMC Plant Biol. 2018 Apr 10;18:57. doi: 10.1186/s12870-018-1278-5 (PMC5892015; doi:10.1186/s12870-018-1278-5)
Supplement: Supplementary file 3 — Table S1. Significantly upregulated genes involved in cell division in transgenic compared to WT plants. (DOCX 15 kb) [file 12870_2018_1278_MOESM3_ESM.docx]

| **No.** | **Gene ID** | **log_2_Ratio**  **(TG vs.WT)** | **Q value** | **Annotation** |
| --- | --- | --- | --- | --- |
| 1 | Pavir. J17007 | Inf | 4.43 E-14 | Cell division cycle protein 48 |
| 2 | Pavir. J39877 | Inf | 2.23 E-02 | Cell division control protein 45 |
| 3 | Pavir. Ha01777 | 9.2312 | 8.08 E-54 | Wall-associated receptor kinase 5 |
| 4 | Pavir. Da02121 | 8.6124 | 3.12 E-38 | Wall-associated receptor kinase 2 |
| 5 | Pavir. J23828 | 7.7392 | 7.09 E-10 | Wall-associated receptor kinase 3 |
| 6 | Pavir. Ia01795 | 5.9397 | 1.64 E-07 | ROOT HAIR DEFECTIVE 3 |
| 7 | Pavir. Da00695 | 5.8594 | 1.70 E-11 | Protein SCHENGEN 3 |
| 8 | Pavir. Ia04615 | 5.4621 | 5.46 E-38 | Elongin 15 kDa subunit |
| 9 | Pavir. Ga00078 | 5.1746 | 3.03 E-04 | S-domain-1 (SD1) receptor kinase 8 |
| 10 | Pavir. Ba00279 | 5.1033 | 3.16 E-06 | COBRA-like protein 7 |
| 11 | Pavir. J22008 | 4.4487 | 4.87 E-04 | Leucine-rich repeat extensin protein 4 |
| 12 | Pavir. J04781 | 3.0480 | 2.47 E-03 | Leucine-rich repeat extensin protein 6 |
| 13 | Pavir. J39558 | 3.3812 | 1.04 E-02 | End-xyloglucan transferase |
| 14 | Pavir. J16678 | 3.6857 | 2.57E-23 | Cell number regulator 8 |
| 15 | Pavir. J20246 | 2.6919 | 1.00 E-04 | Cell number regulator 2 |
| 16 | Pavir. Ha01736 | 2.4668 | 3.88 E-03 | Protein MULTIPLE SPOROCYTE 1 |

**Table S1** Significantly upregulated genes involved in cell division in transgenic compared to WT plants
