# Supplementary material for: Transcriptomic analysis reveals vacuolar Na+ (K+)/H+ antiporter gene contributing to growth, development, and defense in switchgrass (Panicum virgatum L.)
Source: BMC Plant Biol. 2018 Apr 10;18:57. doi: 10.1186/s12870-018-1278-5 (PMC5892015; doi:10.1186/s12870-018-1278-5)
Supplement: Supplementary file 4 — Table S2. List of stress-responsive genes in transgenic compared to WT plants. (DOCX 15 kb) [file 12870_2018_1278_MOESM4_ESM.docx]

| **No.** | **Gene ID** | **log_2_Ratio**  **(TG vs.WT)** | **Function Description** |
| --- | --- | --- | --- |
| 1 | Pavir. J30716 | Inf | Response to abiotic and photooxidative stresses |
| 2 | Pavir. J37501 | 9.3566 | Eestablish immune systemic induced resistance |
| 3 | Pavir. J31186 | 7.1784 | Involved in a defense mechanism |
| 4 | Pavir. J31898 | 6.2218 | A general defense protein; response to stress |
| 5 | Pavir. Aa00547 | 6.9794 | Response to hyperosmotic and heat shock |
| 6 | Pavir. J01404 | 6.6220 | Involved in heat shock signaling pathway |
| 7 | Pavir. J20548 | 6.0802 | 26.2 kDa heat shock protein; response to heat |
| 8 | Pavir. J07818 | 5.3229 | 23.2 kDa heat shock protein; response to heat |
| 9 | Pavir. Ha00186 | 8.5985 | Response to salt stress |
| 10 | Pavir. Ea00535 | 5.0271 | Maintain Na^+^/K^+^ homeostasis under salt stress |
| 11 | Pavir. J16055 | 6.2615 | Response to drought and freezing stress |
| 12 | Pavir. J40048 | 6.6072 | Removal of H_2_O_2_; response to oxidative stress |
| 13 | Pavir. Ba04000 | 5.6998 | Removal of H_2_O_2_; response to oxidative stress |
| 14 | Pavir. Ba01869 | 5.5771 | Repair of DNA lesions; response to oxidative stress |
| 15 | Pavir. Ba00376 | Inf | Involved in heavy metal transport and detoxification |
| 16 | Pavir. Ha00320 | 6.7200 | Plant cadmium resistance protein |
| 17 | Pavir. Ea01215 | 10.5900 | Play a detoxification role against certain herbicides |
| 18 | Pavir. Ia04853 | 9.3242 | Participate in the resistance to certain herbicide |

**Table S2** List of stress-responsive genes in transgenic compared to WT plants
