# Supplementary material for: Transcriptomic analysis reveals vacuolar Na+ (K+)/H+ antiporter gene contributing to growth, development, and defense in switchgrass (Panicum virgatum L.)
Source: BMC Plant Biol. 2018 Apr 10;18:57. doi: 10.1186/s12870-018-1278-5 (PMC5892015; doi:10.1186/s12870-018-1278-5)
Supplement: Supplementary file 5 — Table S3. Significant upregulated transporters in transgenic compared to WT plants. (DOCX 16 kb) [file 12870_2018_1278_MOESM5_ESM.docx]

**Table S3** Significant upregulated transporters in transgenic compared to WT plants

| **Gene ID** | **log_2_Ratio**  **(TG vs.WT)** | | **Q value** | **Annotation** |
| --- | --- | --- | --- | --- |
| **Ion transport** | | | |  |
| Pavir. Eb03651 | | Inf | 2.73 E-05 | Calcium-transporting ATPase1; ECA1 |
| Pavir. Ha00229 | | Inf | 2.27 E-06 | Magnesium/proton exchanger 1; MHX1 |
| Pavir. J05404 | | 8.1504 | 2.79 E-29 | Two pore potassium channel b; KCO2 |
| Pavir. Aa03191 | | 6.1821 | 7.36 E-28 | Aluminum (Al^3+^) transporter; NRAT1 |
| Pavir. Aa03222 | | 6.1548 | 1.16 E-09 | Vacuolar cation/proton exchanger 4 |
| Pavir. J21405 | | 6.0221 | 7.72 E-16 | Anion transporter; POR1 |
| Pavir. J30919 | | 6.0130 | 6.62 E-08 | Calcium-transporting ATPase 5; ACA5 |
| Pavir. Eb03789 | | 5.7887 | 8.07 E-12 | Galcium ion transporter; GLR2.8 |
| Pavir. J02385 | | 5.1012 | 4.78 E-03 | Galcium ion transporter; GLR2.5 |
| Pavir. Cb00042 | | 4.2989 | 1.96 E-02 | Cation/H^+^ antiporter 19; CHX19 |
| Pavir. Gb02254 | | 3.1693 | 2.76 E-08 | Inorganic phosphate transporter; PHT1-13 |
| Pavir. Ib03819 | | 2.6097 | 4.59 E-02 | Potassium transporter 27; HAK27 |
| Pavir. Ea03843 | | 2.4082 | 3.25 E-02 | Potassium transporter 5; HAK5 |
| **Small molecule transport** | | | |  |
| Pavir. Fa02242 | | 6.6712 | 1.09 E-05 | Lysine histidine transporter 1; LHT1 |
| Pavir. Eb02833 | | 6.6411 | 1.14 E-40 | Basipetal auxin transporter; ABCB11 |
| Pavir. Ia02157 | | 6.1983 | 4.80 E-09 | Peptide and histidine transporter; PTR2 |
| Pavir. J03837 | | 6.1599 | 1.13 E-04 | Carbohydrate and sugar transporter; PT505 |
| Pavir. J30972 | | 5.5578 | 1.20 E-05 | Amino acid transporter; PHSC |
| Pavir. Cb01571 | | 2.7226 | 4.22 E-03 | Lysine histidine transporter-like 1; LHTL1 |
| Pavir. Ib04116 | | 2.2909 | 3.22 E-07 | Cationic amino acid transporter 1; CAAT1 |
| **Metal transport** | |  |  |  |
| Pavir. J06378 | | 8.6104 | 5.84 E-31 | Metal-nicotianamine transporter; YSL12 |
| Pavir. Ia01399 | | 6.9565 | 6.35 E-107 | sodium/metabolite cotransporter; BASS1 |
| Pavir. J38980 | | 5.4488 | 5.47 E-04 | Calcium uniporter protein 6; MCU6 |
| Pavir. Ia04441 | | 4.1786 | 2.03 E-04 | Magnesium transporter MRS2-H |
| Pavir. Gb01095 | | 3.0355 | 6.45 E-04 | Copper transporter; HMA5 |
