# Supplementary material for: Transcriptomic analysis reveals vacuolar Na+ (K+)/H+ antiporter gene contributing to growth, development, and defense in switchgrass (Panicum virgatum L.)
Source: BMC Plant Biol. 2018 Apr 10;18:57. doi: 10.1186/s12870-018-1278-5 (PMC5892015; doi:10.1186/s12870-018-1278-5)
Supplement: Supplementary file 6 — Figure S2. Enriched gene ontologies in differentially expressed genes of transgenic switchgrass. Each box shows the GO term number, the p-value in parenthesis, and GO term. Box colors indicates levels of statistical significance: yellow = 0.05; orange = e-05; and red = e-09. (PDF 269 kb) [file 12870_2018_1278_MOESM6_ESM.pdf]

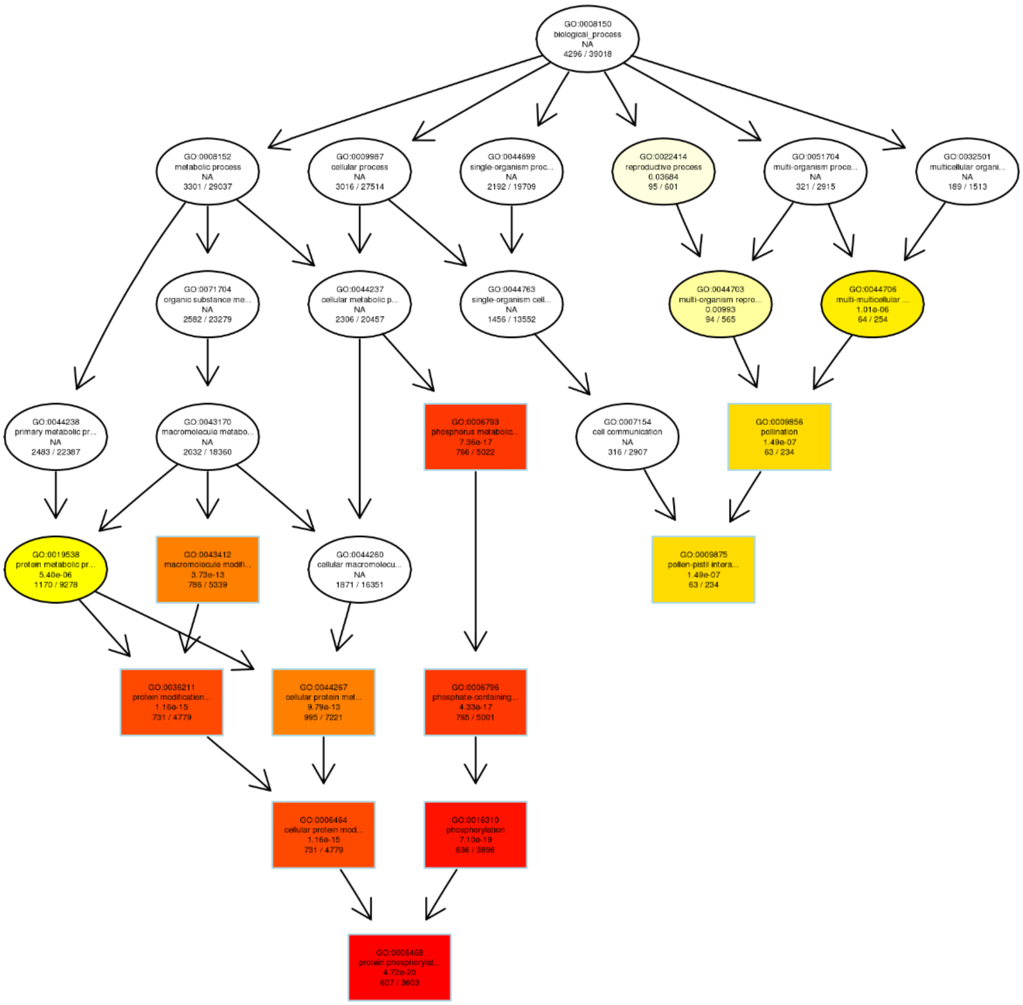

Figure S2. Enriched gene ontologies in differentially expressed genes of transgenic switchgrass. Each box shows the GO term number, the p-value in parenthesis, and GO term. Box colors indicates levels of statistical significance: yellow = 0.05; orange = e-05; and red = e-09.
