# Supplementary material for: Transcriptomic analysis reveals vacuolar Na+ (K+)/H+ antiporter gene contributing to growth, development, and defense in switchgrass (Panicum virgatum L.)
Source: BMC Plant Biol. 2018 Apr 10;18:57. doi: 10.1186/s12870-018-1278-5 (PMC5892015; doi:10.1186/s12870-018-1278-5)
Supplement: Supplementary file 7 — Table S4. Number of genes identified from KEGG pathways. (DOCX 16 kb) [file 12870_2018_1278_MOESM7_ESM.docx]

**Table S4.** Number of genes identified from KEGG pathways

| **No.** | **Term** | **KEGG**  **ID** | **Input number** | **Background number** | **P-Value** |
| --- | --- | --- | --- | --- | --- |
| 1 | Plant-pathogen interaction | sita04626 | 90 | 191 | 4.78 E-03 |
| 2 | Purine metabolism | sita00230 | 76 | 166 | 1.47 E-02 |
| 3 | Glutathione metabolism | sita00480 | 59 | 119 | 9.21 E-03 |
| 4 | Peroxisome | sita04146 | 43 | 93 | 4.99 E-02 |
| 5 | DNA replication | sita03030 | 39 | 54 | 2.62 E-04 |
| 6 | Nucleotide excision repair | sita03420 | 39 | 62 | 1.77 E-03 |
| 7 | Homologous recombination | sita03440 | 38 | 55 | 5.78 E-04 |
| 8 | Aminoacyl-tRNA biosynthesis | sita00970 | 30 | 59 | 4.19 E-02 |
| 9 | Porphyrin and chlorophyll metabolism | sita00860 | 29 | 43 | 3.35 E-03 |
| 10 | Mismatch repair | sita03430 | 27 | 39 | 1.47 E-02 |
| 11 | Valine, leucine and isoleucine biosynthesis | sita00290 | 11 | 15 | 4.07 E-02 |
