# Supplementary material for: Transcriptomic analysis reveals vacuolar Na+ (K+)/H+ antiporter gene contributing to growth, development, and defense in switchgrass (Panicum virgatum L.)
Source: BMC Plant Biol. 2018 Apr 10;18:57. doi: 10.1186/s12870-018-1278-5 (PMC5892015; doi:10.1186/s12870-018-1278-5)
Supplement: Supplementary file 8 — Figure S3 Differentially expressed transcription factors in transgenic switchgrass. Blue bar represents down-regulated DEGs; red bar indicates up-regulated DEGs. (PDF 133 kb) [file 12870_2018_1278_MOESM8_ESM.pdf]

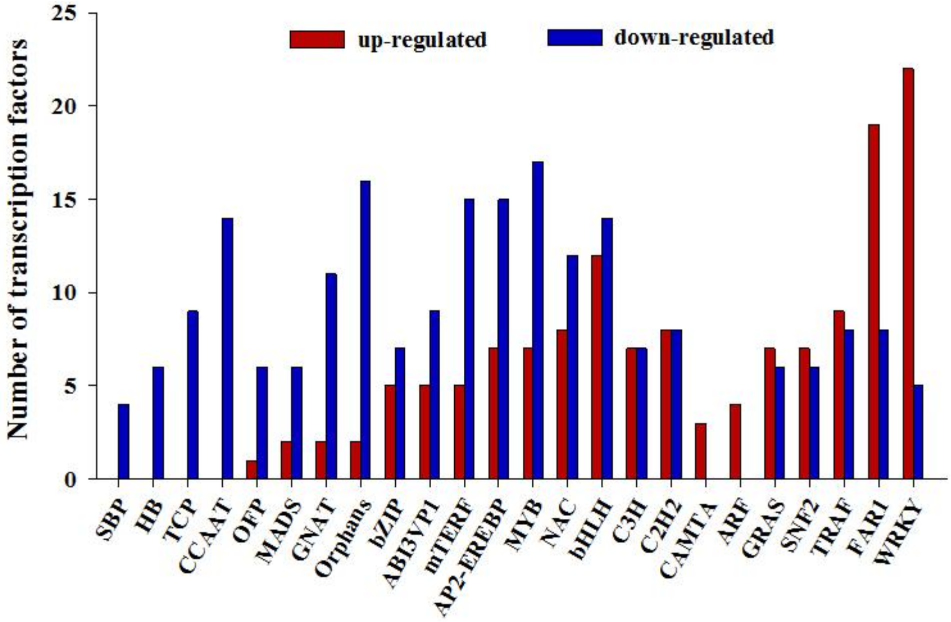

Figure S3. Differentially expressed transcription factors in transgenic switchgrass. Blue bar represents down-regulated DEGs; red bar indicates up-regulated DEGs.
