# Supplementary material for: Transcriptomic analysis reveals vacuolar Na+ (K+)/H+ antiporter gene contributing to growth, development, and defense in switchgrass (Panicum virgatum L.)
Source: BMC Plant Biol. 2018 Apr 10;18:57. doi: 10.1186/s12870-018-1278-5 (PMC5892015; doi:10.1186/s12870-018-1278-5)
Supplement: Supplementary file 9 — Figure S4 Expression analysis of selected RNA-seq genes by qRT-PCR. FPKM (fragments per kilobase of exon per million fragments mapped) values obtained with RNA-seq and qPCR values in the analysis of selected genes. Error bars represent the standard error for three independent experimental replicates. (PDF 146 kb) [file 12870_2018_1278_MOESM9_ESM.pdf]

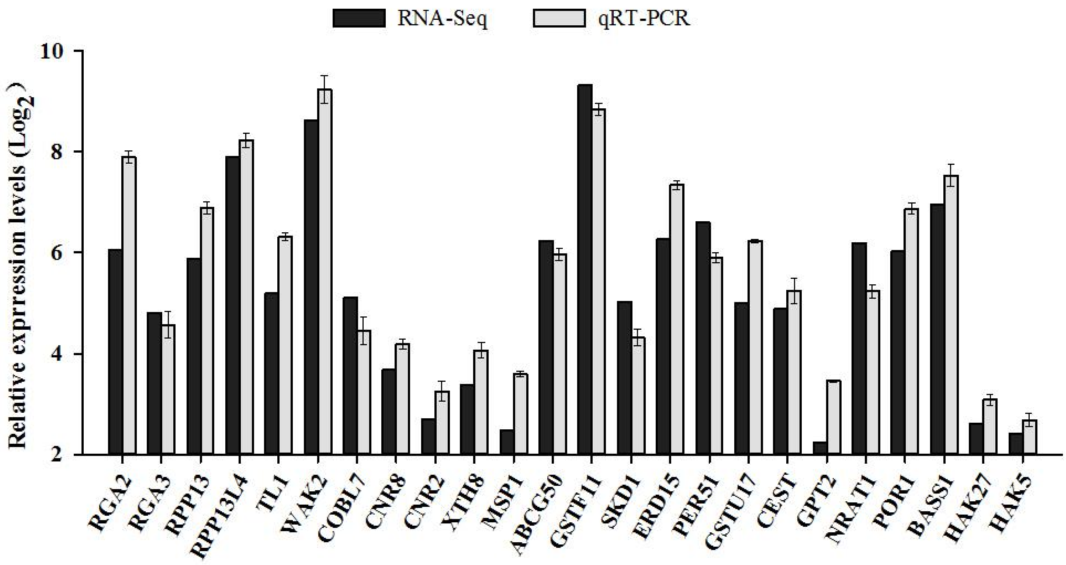

Figure S4. Expression analysis of selected RNA-seq genes by qRT-PCR.

FPKM (fragments per kilobase of exon per million fragments mapped) values obtained with RNA-seq and qPCR values in the analysis of selected genes. Error bars represent the standard error for three independent experimental replicates.
